# Smoking under hypoxic conditions: a potent environmental risk factor for inflammatory and autoimmune diseases

**DOI:** 10.1186/s40779-018-0158-5

**Published:** 2018-03-30

**Authors:** Md. Saddam Hussain, Vishwas Tripathi

**Affiliations:** School of Biotechnology, Gautam Buddha University, Greater Noida, Gautam Budh Nagar, Uttar Pradesh 201312 India

**Keywords:** Inflammatory and autoimmune diseases, Cigarette smoke, Hypobaric hypoxia, Oxidative stress, Epigenetic modifications, Pro-inflammatory mediators, Autoantibody

## Abstract

Autoimmune disease management presents a significant challenge to medical science. Environmental factors potentially increase the risk of developing inflammatory and autoimmune diseases, such as multiple sclerosis, rheumatoid arthritis, and lupus. Among various environmental stresses, cigarette smoke and hypoxia have both been reported to lead to an enhanced risk of inflammatory and autoimmune diseases.

In this review, we shed light on all reported mechanisms whereby cigarette smoke and a hypoxic environment can induce inflammatory and autoimmune diseases and discuss how hypoxic conditions influence the cigarette smoke-induced threat of inflammatory and autoimmune disease development.

Cigarette smoke and hypoxia both lead to increased oxidative stress and production of reactive oxygen species and other free radicals, which have various effects including the generation of autoreactive pro-inflammatory T cells and autoantibodies, reductions in T regulatory (T_reg_) cell activity, and enhanced expression of pro-inflammatory mediators [e.g., interleukin-6 (IL-6), interleukin-4 (IL-4) and interleukin-8 (IL-8)]. Accordingly, smoking and hypoxic environments may synergistically act as potent environmental risk factors for inflammatory and autoimmune diseases. To our knowledge, no studies have reported the direct association of cigarette smoke and hypoxic environments with the risk of developing inflammatory and autoimmune diseases.

Future studies exploring the risk of autoimmune disease development in smokers at high altitudes, particularly military personnel and mountaineers who are not acclimatized to high-altitude regions, are required to obtain a better understanding of disease risk as well as its management.

## Background

Autoimmune disease has been a hot topic in medical science for the last decade. Autoimmunity is the state of the immune system in which immune cells recognize self-molecules as antigens and become hyperactive to eliminate these molecules, resulting in chronic inflammation, pain and various other severe conditions. Currently, a major portion of the world population [1] suffers from various types of autoimmune diseases, including rheumatoid arthritis (RA), systemic lupus erythematosus (SLE), multiple sclerosis (MS), thyroid disease, inflammatory bowel disease (IBD), Graves’ disease (GD), and Behcet’s syndrome. The major challenges of autoimmune diseases are to decipher their precise causes and mechanisms to develop more effective and efficient treatments.

Environmental factors play crucial roles in the development and progression of inflammatory and autoimmune diseases. Among such factors, cigarette smoke (CS) and hypoxia are two potent environmental stresses that can significantly cause imbalance in normal immune homeostasis by modulating immune-regulatory activities, which may lead to inflammatory and autoimmune diseases.

Despite widespread knowledge of the health risks posed by tobacco consumption in the form of smoke and other means, consumption of tobacco remains unacceptably prevalent [2]. Tobacco smoking killed almost 6 million people worldwide in 2011, which include about 600,000 people that never smoke and died due to indirect second-hand smoke. If it follows the same way, the death number will reach to 800,000 per year by 2030, higher than the mortality resulting from human immunodeficiency virus and acquired immune deficiency syndrome (HIV/AIDS), tuberculosis and malaria combined. Indeed, the threat of tobacco smoking to public health can be considered to be a worldwide epidemic [3–7]. CS can lead to inflammatory and autoimmune diseases through multiple mechanisms, including the following: genetic/epigenetic modifications; increased oxidative stress, reactive oxygen species (ROS), and free radical production, and nicotine and heavy metal toxicity. These effects, in turn, may increase B and T cell proliferation, reduce immune suppressive T regulatory (Treg) cell proliferation and activity as well as autoantibody generation, and enhance expression of pro-inflammatory mediators, such as IL-6, IL-8, tumor necrosis factors (TNFs) and Interferon gamma (INF-γ). Hence, CS is a risk factor for developing inflammatory and autoimmune diseases.

Decreased oxygen availability (hypobaric hypoxia at high altitude or cellular hypoxia) can also alter the normal function of the immune system in multiple ways, making it to be more susceptible to various inflammatory and autoimmune diseases. Recent advancements in our understanding of oxygen-dependent cell signaling have revealed several mechanisms by which hypoxia impacts the development of inflammation through the coordinated expression of inflammatory, adaptive and apoptotic genes. Hypoxia is a distinctive microenvironmental feature in a number of inflammatory conditions, including IBD and arthritis [8–10]. Hypoxia induces increased expression of hypoxia-inducible factor (HIF), resulting in overexpression of pro-inflammatory mediators, and also causes enhanced oxidative stress, which results in autoantibody generation and transformation of CD4^+^ T cells into auto-reactive pro-inflammatory cells.

In this review, we highlight the effects of smoking and hypoxic conditions (hypobaric hypoxia and cellular hypoxia) on the immune system and focus on different ways by which these conditions increase the risk of developing inflammatory and autoimmune diseases. To our knowledge, the combined effect of smoking and hypoxia on the immune system and the subsequent risk of developing autoimmune diseases have not yet been reviewed. Therefore, we summarize all available information concerning the association of smoking and hypoxia with the risk of developing various inflammatory autoimmune diseases. Based on the findings, we propose that the combination of smoking and hypoxic conditions may act as a potent environmental risk factor for inflammatory and autoimmune disease development. Future studies are needed to explore the risk of the development of autoimmune diseases in smokers in high-altitude regions, specifically military personnel and mountaineers who are not acclimatized to high-altitude stresses. These findings may also help to enhance our current understanding and management of human health in high-altitude regions.

## CS and autoimmune diseases: overview

Tobacco consumption, either in the form of CS or other means, is theoretically an avoidable environmental factor yet a major cause of various health issues and death worldwide [5–7]. CS contains thousands of complex, dynamic, and reactive chemical constituents that possess cytotoxic, mutagenic, immune-modulators and tumorigenic properties [11–23]. Several toxic components of CS have immunomodulatory effects that result from genetic/epigenetic changes and lead to altered gene expression and function; some examples include changes in pro-inflammatory cytokine expression and histone deacetylase (HDAC) and histone acetylase (HAT) activities [2]. Several studies have demonstrated CS to be a potent environmental risk factor for certain autoimmune diseases, such as RA, MS, and SLE [24–26]. Since the first evidence provided in 1987 by Vessey and colleagues [27] that CS increases the risk of RA development (approximately 2.4 times elevated risk among women smokers) until 2006, 11 case-control and 4 cohort studies have been reported, showing an increased risk of RA development in cigarette smokers (Table 1) [24, 26–40]. In fact, a twofold increased risk of developing seropositive RA for individuals who smoked for more than 20 years and a threefold increased risk for RA in male smokers have been reported [2, 41–43]. In addition, Freemer and coworkers conducted a case-control study showing a link between CS and SLE; these authors reported the presence of an increased level of anti-dsDNA antibodies in current smokers compared to former-and non-smokers with SLE (Table 1) [42]. Furthermore, the association of CS with an increased risk of MS is also evident based on the results of various epidemiological studies [44–48] (Table 1, [24–68]).

**Table 1:** Reported epidemiological studies of the relationship between CS and the risk of selected autoimmune diseases

Autoimmune disease	Case-control studies (No. of studies showing increased risk/total no. of studies)	Cohort studies (No. of studies showing increased risk/total no. of studies)	Range of observed *OR (RR)* of developing diseases
RA	11/12 [24, 26–28, 31–34, 36–39]	4/4 [29, 30, 35, 40]	0.6-3.4 -Risk increases with increases in the duration and intensity of smoking -Males are more prone
MS	1/3 [44–46]	2/2 [47, 48]	1.6-1.9 -Risk increases with increases in the intensity of smoking
SLE	3/8 [49–56]	0/2 [57, 58]	0.5-6.7 -Current smokers are mainly at risk
GD	8/8 [42, 59–65]	1/1 [66]	1.3-8.2 -Current smokers are at higher risk than former smokers -Risk increases with increases in the intensity of smoking -Females might be more prone
Primary biliary cirrhosis	2/2 [67, 68]	-	1.6-3.5

*RA* Rheumatoid arthritis, *MS* Multiple sclerosis, *SLE* Systemic lupus erythematosus, *GD* Graves’ disease, *-* No data

## CS and associated genetic changes

Somatic mutations are reported to be crucial factors in the pathogenesis of autoimmunity. To induce autoimmunity, coherent type-a somatic mutations are required, which occur in multiple cells to such an extent that somatically mutated proteins lose their normal functions and/or are interpreted by the immune system to be non-self, i.e., autoantigens [69]. Simple tandem repeats (STRs) within protein coding genes, including microsatellites and mini-satellites are highly vulnerable regions that are susceptible to mutations in somatic and germ-line cells, and somatic mutations in these STRs generate novel, highly immunogenic proteins [70, 71] that may induce autoimmunity. Moreover, repetitive sequences play a crucial role in methylating nearby sequences [72]. Indeed, mutations in repeat sequences often result in significant changes in methylation patterns [73], which in turn alter normal splicing [74], and altered methylation has been associated with several autoimmune diseases [75]. Overall, the mutability of tandem repeats intensifies with increasing repeat length, repeat count, and high repeat identity [76]. Long STRs within 20 genes have been associated with 16 common autoimmune diseases; these genes include the following: thyroid peroxidase (TPO), which encodes a primary autoantigen in Hashimoto’s thyroiditis (HT) and Grave’s disease (GD); filaggrin (FLG), which encodes a primary autoantigen in RA; and protein-tyrosine phosphatase, receptor-type, n, polypeptide 2 (PTPRN2), which encodes a primary autoantigen in type-1 diabetes (T1D) (see Table 7 of reference [76]). Single-nucleotide polymorphisms (SNPs) are also associated with the pathogenesis of autoimmune diseases. The functional SNP rs2476601 in the PTPN22 gene has been linked to many autoimmune diseases, though no association has been found for MS, pernicious anemia (PA), and Sjogren’s syndrome (SJ) (see Table 1 of reference [76]). This SNP specifically affects T cell signaling [77, 78], B cell signaling [79, 80]; causes autoreactive B cell generation [79], and T cell and dendritic cell hyper-responsiveness [81].

Exposure to CS, directly (mainstream tobacco smoke; MTS) or indirectly (second-hand smoking), is mutagenic, and MTS is considered to be the most extreme example of a human systemic mutagen [82]. CS contains more than 4,000 chemical constituents [83], many of which are genotoxic and interact with DNA to induce mutations and gene activation, leading to the development of autoimmune diseases [82, 84]. Somatic mutations result in autoimmunity, and CS induces both somatic and germ-line mutations [82, 85]. Evidence of CS-induced somatic mutations is well reported [82, 86, 87]. For example, the frequency of somatic hypoxanthine-guanine phosphoribosyl transferase (HPRT) gene mutations is significantly elevated in both adult smokers [88] and newborns of smoking mothers [89]. CS has also been specifically associated with the production of autoantibodies against primary autoantigens encoded by mutated enolase [90], vimentin [91], and fibrinogen beta [91] genes in RA. Although elevated levels of DNA adducts, strand breaks and oxidative damage have been reported in the sperm of male smokers [82, 92], evidence for MTS-induced heritable germ-line mutations is lacking. In 2007, Yauk et al. [93] reported that MTS induces a significantly elevated frequency of germline mutations at the expanded simple tandem repeat (ESTR) locus *Ms6-hm* in mouse sperm, which could, at least theoretically, be inherited in subsequent generations.

## Epigenetic modifications induced by CS

All known epigenetic pathways are altered by both active and passive (including in utero) CS and such epigenetic changes can be transmitted to the next generation through the male germ line [94]. Such epigenetic alterations caused by CS include post-translational modifications through HAT, HDAC, and DNA methyl transferase (DNMT), leading to chromatin remodeling and changes in gene expression [95].

### Histone acetylation/deacetylation

Reversible acetylation/deacetylation of conserved lysine residues (at α-amino groups) in the histone tails of chromatin has an important function in regulating gene transcription [96]. Acetylation of histones promotes gene expression by facilitating the recruitment of transcription factors, such as NF-κB, and enabling access of the transcriptional machinery to DNA. Conversely, deacetylation of histones negatively regulates transcription by decondensing the chromosome and decreasing the accessibility of transcription factors, including NF-κB and AP-1, to their respective DNA binding motifs [95, 97–99].

HAT and HDAC are responsible for acetylating and deacetylating, respectively, lysine residues on histone and non-histone proteins. To date, 18 isoforms of HDAC have been identified in humans named as HDAC-1 to HDAC-11 and SIRT-1 to SIRT-7 which are further classified into four major groups namely class 1 to class 4 [100–103]. The addition of acetyl groups to histone lysine residues by HATs cause unwinding of chromatin and hence transcriptional activation of genes. In contrast, HDACs function as transcriptional co-repressors by removing acetyl groups, which results in chromosomal condensation, exclusion of transcription factors and, eventually, inhibition of gene transcription. In addition to histones, HDACs also deacetylate non-histone proteins, such as NF-κB, and regulate NF-κB-dependent pro-inflammatory gene transcription [95, 102]. Indeed, HATs and HDACs play significant roles in the immunological balance, and alterations in the functions of these proteins lead to immunological disorders. Furthermore, HDAC-1, -3, -6, -9 and SIRT-1 also act as inhibitors of T_reg_ cell development and inhibit their immune-suppressive function by deacetylating FOXP3 (a bona fide marker of active T_reg_ cells), whereas HATs [p300, TIP60, and PCAF (p300/CREB binding protein-associated factor)] acetylate FOXP3 and positively regulate T_reg_ cell development and function [104, 105]. As T_reg_ cells negatively regulate autoimmune processes, inhibition of T_reg_ cell function and development as a result of enhanced HDAC function and/or HAT inhibition potentially lead to inflammatory and autoimmune diseases.

**Fig. 1:**
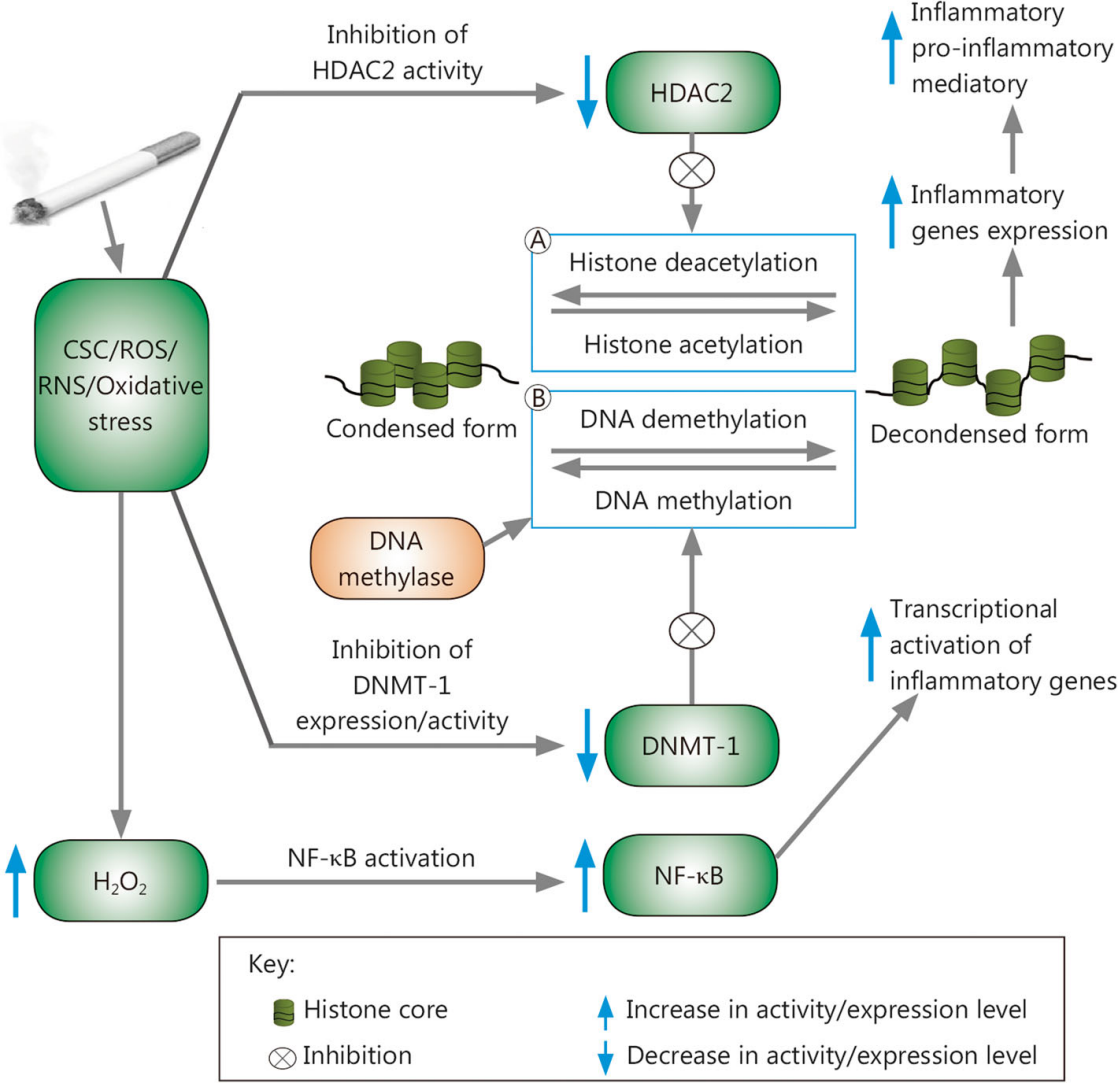
Epigenetic modifications induced by cigarette smoke. **a** Acetylation: *via* a decrease in HDAC2 activity and NF-κB activation, cigarette smoke induces an increase in histone-4 acetylation and hence up-regulation of inflammatory gene expression. **b** DNA methylation: in mammals, the cytosine in CpG motifs (not shown in the figure) can be reversibly methylated at the 5′ position by DNMT; CSC and ROS/RNS produced in cigarette smoke reduce DNMT-1 expression/activity, inhibiting DNA methylation and hence enhancing expression of methylation-regulated inflammatory genes, which leads to inflammatory and autoimmune diseases. CSCs. cigarette smoke constituents; DNMT. DNA methyl transferase; HDAC2. histone deacetylase-2; NF-κB. nuclear factor-kappa B; ROSs. reactive oxygen species; RNS. reactive nitrogen species

CS causes imbalance in the normal histone acetylation/deacetylation process, leading to sustained transcription of pro-inflammatory protein genes by inhibiting HDAC activity and activating NF-κB and AP-1, which in turn results in chronic inflammation (Fig. 1a) [106]. Moreover, CS stimulates oxidative stress by inducing generation of ROS, H_2_O_2_ and free radicals. HDAC2 activity is significantly reduced in response to smoking-induced oxidative stress, and alteration of HDACs by CS exposure leads to histone acetylation and SIRT1 post-transcriptional modifications and subsequent degradation; this disrupts the co-repressor complex (SIRT1-RelA/p65 complex) and leads to activation of redox-sensitive transcription factors such as NF-κB, increasing transcription of pro-inflammatory genes encoding IL-8, IL-6, and TNFs [2, 96, 106–114].

HDAC inhibition and HAT activation through cigarette smoke constituents (CSCs) and the increased oxidative stress induced by CS result in higher Nrf2 acetylation, which destabilizes the protein. These effects reduce anti-oxidant gene expression and increase sensitivity to oxidative stress, making individuals more prone to chronic obstructive pulmonary disease (COPD)-like inflammatory and autoimmune diseases [96, 115].

### DNA methylation/demethylation

DNA methylation, an important heritable epigenetic change that occurs at cytosine residues in CpG motifs, stabilizes chromatin in a tightly packed conformation, thereby suppressing gene expression [108]. DNA methylation and demethylation are catalyzed by a specific group of enzymes, DNMTs and DNA methylases, respectively. These enzymes are sensitive to environmental factors; for example, oxidative stress due to cigarette smoking can result in reduced DNMT-1 activity and thus increased DNA demethylation [116].

Studies have shown that CSCs and ROS/reactive nitrogen species (RNS), e.g., H_2_O_2_/NO_3_, inhibit the extracellular *signal*-regulated kinase (ERK) signaling pathway in T cells, leading to a reduction in DNMT-1 expression and thus reduced DNA methylation. This effect, in turn, results in chromatin remodeling, leading to increased expression of methylation-regulated genes (Fig. 1b) that contribute to lupus flare-like autoimmune disease. DNMT-1 inhibition due to CS-induced oxidative stress increases expression of *lymphocyte function-associated* antigen-a heterodimer composed of *CD11a* and *CD18 [*LFA (CD11a/CD18)] and CD70 in T cells and changes antigen-specific CD4^+^ T helper cells into auto-reactive pro-inflammatory cells, which respond to self-class 2 MHC molecules without added antigens and kill autologous macrophages, resulting in lupus-like autoimmunity [108, 116].

### Histone phosphorylation

Although information regarding phosphorylation of histones is limited, it is clear that these molecules play significant roles in chromatin conformation and thereby regulate gene transcription. Studies have shown that phosphorylation of histone H3 facilitates its acetylation, which involves cAMP-response element-binding protein (CREB-binding protein) [117]. Cigarette smoke results in increased levels of phosphorylated (Ser10) and acetylated (Lys 9) histone H3, which leads to increased pro-inflammatory cytokine release by macrophages and mouse lungs [118]; these events indicate a potential risk of inflammatory and autoimmune diseases.

## CS and autoantibody production

Many studies have reported an association between CS and the production of autoantibodies, such as anti-citrullinated peptide antibodies and autoantibodies against elastin [2]. Increased sero-positivity for dsDNA (i.e., anti-dsDNA antibodies) has been found in current smokers relative to former smokers and serves as a diagnostic marker for SLE [42, 119]. A lack of methylation or demethylation leads to anti-dsDNA antibody production, which sufficiently induces lupus-like autoimmunity in genetically predisposed mice and, likely, humans [108]. Oxidative stress caused by CS is a potential risk factor for autoantibody production. For example, elevated ROS in red blood cells (RBCs) results in the production of autoantibodies against RBCs and causes autoimmune hemolytic anemia (AIHA) [117].

## CS and heavy metals

The filler tobacco used in cigarettes of different brands contains Cd (Cadmium), Ni (Nickel) and Pb (Lead) at concentrations that range 1.73-2.02, 0.715-1.52, and 0.378-1.16 μg per cigarette, respectively, and this increased exposure of toxic metals due to CS may increase RA risk [43]. For instance, oral exposure to Cd at environmental levels has been associated with the increased production of autoantibodies, which may be due to dose-sensitivity and polyclonal B cell activation (PBA) [120], and Pb exposure via CS increases the risk of MS [121].

Ni and Cd strongly activate NF-κB and induce the release of IL-8 in the THP-1 human monocytic leukemia cell line. Cadmium also induces TNF-α and IL-6 release in THP-1 monocytic cells. Hence, sustained activation of transcription factors (e.g., NF-κB) by metal-activated signaling may lead to chronic inflammatory processes and related diseases, such as autoimmune disorders [122]. Moreover, nicotine has been associated with autoimmune arthritis and may also differentially affect the severity of rodent autoimmune arthritis [2].

## CS and altered function of immune system

The reported potential mechanisms by which CS promotes RA include augmentation of autoreactive B cells functions [38], altered antigen presentation by the CS-impaired *antigen* presentasome (APS) [123, 124], changes in T_reg_ cell functions (also promoting COPD) [125] and activation and proliferation of T cells by antigens present in CS. Furthermore, CS significantly reduces the cytotoxic activity of natural killer (NK) cells [126], which destroy auto-reactive T cells that promote autoimmune disease [127], and reduces the production of IFN-γ and TNF-α by activated NK cells [126]. Another study reported that CS also induces polyclonal activation of both B and T cells, enhances the production of several cytokines (e.g., IL-2, IL-4, and soluble ICAM-1) while reducing that of IFN-γ, increases serum IgE levels, and enhances antigen presentation by damaging cells. This evidence suggests a potential link between CS and the development and an increased risk for inflammatory and autoimmune diseases, including RA, Good pasture syndrome, Grave’s ophthalmopathy and autoimmune hypothyroidism [128].

T cells may be induced to proliferate into different types of inflammatory cells, categorized as Th1, Th2, and Th17 type inflammation-inducing and respective pro-inflammatory cytokine-producing T cells [129]. Compared to non-smokers, smokers have enhanced IL-13 (Th2 cytokine) levels and reduced levels of Th-1 cytokines; hence, CS may be considered to be an environmental agent that induces Th2 type inflammation or acts as an adjuvant of adaptive Th2 immunity [124, 130–134]. Available evidence also suggests that in certain individuals, chronic CS exposure causes Th17 inflammation-associated inflammatory diseases and may also promote adaptive Th17 immunity to self-antigens [24, 135, 136]. Moreover, CS-induced oxidative stress increases expression of LFA (CD11a/CD18) and CD70 in T cells and changes antigen-specific CD4^+^ T helper cells into autoreactive pro-inflammatory cells, which respond to self-class 2 major histocompatibility complex (MHC) molecules without added antigens and kill autologous macrophages, potentially causing lupus-like autoimmunity [108, 116].

## Hypoxic conditions and inflammatory disorders

**Fig. 2:**
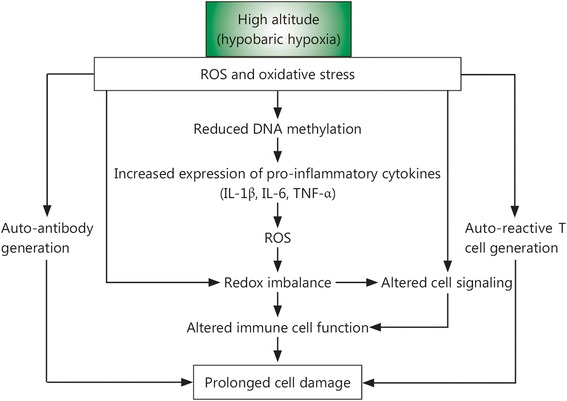
Potential mechanisms of hypobaric hypoxia-induced inflammation. Hypobaric hypoxia enhances the ROS level in body beyond the limit of tolerance, which further induces oxidative damage and alters the immune cell functions. The increased oxidative stress in turn heightens the expression of pro-inflammatory cytokines, generation of autoantibodies (e.g., anti-dsDNA, anti-elastin and anti-RBC autoantibodies) and conversion of T helper cells into auto-reactive T cells, eventually results in persistence inflammation and prolonged cell damage. ROS, Reactive oxygen species; IL, Interleukin

Environmental conditions potentially challenge the capacity of the human body’s defense network. Hypoxia associated with high altitude, which is typically regarded as an elevation over 2,400 m, is an important environmental assault. For example, if the O_2_ concentration at sea level is accepted to be 100%, this percentage gradually decreases with increasing elevation. Thus, the decreased O_2_ availability at high altitude, i.e., hypobaric hypoxia, due to reduced O_2_ pressure results in increased formation of ROS and RNS (RONS), a process that is linearly related to the elevation, degree of hypoxia and duration of exposure. In these cases, the ROS levels exceed the defense response of the body, which in turn enhances oxidative damage to macromolecules such as lipids, proteins, and DNA as well as the entire cell (Fig. 2). Exposure to hypobaric hypoxia also significantly reduces the activity and effectiveness of both enzymatic and non-enzymatic antioxidant systems. Moreover, during high-altitude exposure, different RONS-producing sources, including the mitochondrial electron transport chain, xanthine oxidase, and nitric oxide synthase (NOS), gets activated; UV radiation is also strongly increased, leading to overproduction of ROS [137–140]. Elevated ROS levels may lead to or increase the risk for inflammatory and autoimmune diseases through heightened autoantibody production, including anti-dsDNA antibodies, and pro-inflammatory gene expression, and the generation of auto-reactive T cells.

Among the various transcription factors that facilitate cellular adaptation to hypoxic environments (e.g., hypobaric hypoxia or cellular hypoxia), HIF-1 is one of the most important. HIF-1 was first reported in a nuclear extract of the Hep3B human hepatoma cell line [141] and subsequently described as a heterodimeric protein comprising HIF-1α and -1β subunits. The β subunit is constitutively expressed, whereas α subunit is stabilized in the absence of oxygen. There are three isoforms of α subunit, HIF-1α, HIF-2α and HIF-3α, which are distinguished by the presence of *basic helix-loop-helix* (bHLH), Per-ARNT-Sim (PAS) and oxygen-dependent degradation (ODD) domains. Despite the many structural and functional similarities, HIF-1α is ubiquitously expressed, whereas HIF-2α is tissue specific and, in certain cases mediates different biological functions [142]. HIF-2α isoforms have also been implicated in cartilage destruction: in an arthritis mouse model, HIF-2α induced chemokine expression in chondrocytes, which stimulated the migration and invasion of synovial fibroblasts, leading to cartilage erosion [143]. Recent studies have shown that HIF-2α plays a fundamental role in RA, independent of HIF-1α. Under hypoxic conditions, HIF-1α expression is enhanced via inactivation or reduced activity of prolyl hydroxylase (PHD) enzymes, which degrade and destabilize HIF-1α under normoxic conditions through oxidation of the ferrous ion at the active site [144, 145]. Under normoxic conditions, PHD catalyzes hydroxylation of proline residues in the ODD domains of HIF-1α. The tumor suppressor von Hippel Lindau (vHL) protein subsequently recognizes these hydroxylated residues and recruits the ubiquitin ligase complex, leading to poly-ubiquitination and eventual proteasomal degradation of HIF-1α [146]. Asparagyl β-hydoxylases, also called factor-inhibiting HIF-1 (FIH-1), can also suppress HIF-1α activity by hydroxylating asparagine residues in the C-terminal trans-activation domain, thereby preventing HIF-1α interaction with its co-activator [147–149]. As these oxygen-dependent modifications do not occur under hypoxia, HIF-1α does not bind to vHL, resulting in stabilization and accumulation of HIF-1α in the cytoplasm, followed by its translocation to the nucleus. Inside the nucleus, HIF-1α dimerizes with the HIF-1β subunit, forming the HIF-1αβ heterodimer that binds to the promoter region of HIF-regulated genes [150]. Cramer et al. [8] reported the first evidence of the involvement of HIF-1α in the inflammatory process, showing that HIF-1α deletion in macrophages reduces the severity of disease in different models of acute and chronic inflammation, including a passively induced arthritis disease model. HIF-1α also promotes signaling pathway activation and regulates IL-33 production by fibroblasts, which in turn induces expression of HIF-1α and forms a regulatory mechanism that perpetuates inflammation in RA [151]. Loss or inhibition of HIF-1α function enhances T_reg_ cell differentiation and function [150, 152]. Thus, hypoxic conditions that increase the synthesis of the HIF-1α protein, a negative regulator of FOXP3 (a bona fide marker of T_reg_ cell) expression, significantly reduce the development and activity of T_reg_ cells, thereby increasing the risk of inflammatory and autoimmune diseases.

HIF-1α is inhibited by HDAC [153], and smoking, as noted above, is an inhibitor of HDAC [2]. Thus, smoking under hypoxic conditions leads to increased HIF-1α activity, which in turn reduces the proliferation and activity of T_reg_ cells. Hence, smoking in a hypoxic state is a potent risk factor for various inflammatory and autoimmune diseases. Hypoxia-induced HIF-1α expression increases the maturation of dendritic cells, which results in inflammatory immune responses, e.g., renal injury [154].

Furthermore, HIF-1 regulates the Th17/ T_reg_ balance, and studies have shown that the imbalance between the number and function of Th17 and T_reg_ cells may lead to inflammatory and autoimmune diseases, such as IBD and RA, and impact their severity. During hypoxia, HIF-1 promotes Th17 differentiation, prevents apoptosis and inhibits T_reg_ cell differentiation; down-regulation and/or dysfunction of T_reg_ cells promote autoimmune diseases and inflammation [155].

In addition to HIF, the key cellular response to hypoxia, nearly 20 different transcriptional factors that directly or indirectly sense hypoxic microenvironments have been identified [156]. The principle factors are members of the NF-κB family, the activation of which has been extensively reported in many cell types in response to hypoxia [156, 157], which are also the main pro-inflammatory transcription factors. Individuals exposed to hypobaric hypoxia show increased activation of NF-κB. Under normoxic conditions, PHDs hydroxylate and inhibit IKK activity, which does not occur under hypoxic conditions, enabling IKK-mediated phosphorylation and promoting degradation of IκB and thus activating NF-κB. NF-κB further activates expression of various pro-inflammatory cytokines, chemokines, TNF-α, IL-6, IL-8, vascular endothelial growth factor (VEGF), matrix metalloproteinase (MMP) 1, -3, and -13, and many other proteins that lead to the activation of a positive feedback loop, which enhances activation of more pro-inflammatory signals and eventually results in chronic and persistent inflammation, tissue destruction [158–161] and autoimmune diseases. Abnormal activation of NF-κB has been associated with inflammation-related diseases, particularly RA and IBD [162]. Moreover, hypoxia is a characteristic of RA synovial tissue [163–166], and NF-κB is overexpressed in this tissue [159]. In RA, hypoxia might play a role in sustaining and inducing inflammation, consistent with the study of Jeon and colleagues [167], who used a murine model of collagen-induced arthritis (CIA). Synovial fibroblasts in RA patients express an endogenous TLR ligand called high-mobility group box 1 (HMGB-1), which up-regulates expression of VEGF, thereby exacerbating RA [168], though inhibition of HIF-1α leads to attenuation of the HMGB-1 protein. Hypoxia and IL-17 increase expression of MMP2 and MMP9 via the NF-κB-HIF-1α pathway and thus synergistically promote the migration and invasion of synovial fibroblasts in RA [169]. RA synovial fibroblasts exposed to hypoxia have increased levels of MMPs (MMP1 and MMP3) and decreased levels of tissue inhibitors of MMP1 (TIMP-1) at both the mRNA and protein levels, promoting the destruction of articular cartilage in these patients [170, 171]. The hypoxia-inducible transcription factor Ets-1 [172] has also been implicated in the invasion and destruction of cartilage and bone in RA [173].

In addition to acting independently in the regulation of gene expression in hypoxic inflammation, HIF and NF-κB also exhibit a significant level of crosstalk. NF-κB plays an important role in the up-regulation of HIF-1α mRNA expression [174–176], and HIF-1α can also modulate NF-κB signaling. A previous study showed that mice overexpressing HIF-1α in keratinocytes had increased activity and expression of NF-κB, which regulates pro-inflammatory and anti-apoptotic genes, resulting in hyper-responsiveness to inflammatory stimuli [177].

However, it remains unclear whether HIF-1α acts as an inflammatory or anti-inflammatory factor. As HIF-1α promotes the survival of inflammatory cells [8], it can be considered to be pro-inflammatory. In contrast, expression of HIF-1α in intestinal epithelial cells contributes to an intestinal epithelial barrier function in cases of inflammation, and the intestinal epithelial barrier prevents non-specific movement of luminal antigenic substances into the sub-epithelial lamina propria. Thus, in this context, the HIF pathway can be considered anti-inflammatory [178]. A previous study reported that overexpression of HIF-2α increased inflammation and RA, whereas overexpression of HIF-1α did not [145]. NF-κB activation under hypoxic conditions stabilizes HIF-1α, thereby activating the HIF-1 transcription factor. Another study showed that prolonged HIF activation in healthy individuals living at high altitudes might reduce NF-κB activity, effectively dampening immune responses [145]. Thus, the role of NF-κB in hypoxia-induced inflammatory and autoimmune diseases requires further investigation.

A glycolytic shift is another mechanism by which hypoxia promotes inflammatory and autoimmune diseases. Glycolysis serves as a key metabolic checkpoint to regulate cell fate determination between Th-17 and T_reg_ cells. Hypoxic conditions induce a metabolic shift toward glycolysis [179], enhancing expression of HIF-1α and activation, growth and proliferation of Th-1, Th-2, and Th-17 cells and also inhibit T_reg_ cells. Conversely, blocking glycolysis inhibits Th-17 development while promoting T_reg_ cell generation [152]. Under hypoxic conditions, HIF-1α up-regulates expression of the glucose transporters GLUT1 and GLUT3 to enhance glucose uptake and controls expression of hexokinase II, glyceraldehyde 3-phosphate dehydrogenase, lactate dehydrogenase, and mitochondrial cytochrome oxidase to increase the glycolysis rate in RA synovial tissue as well as expression of glucose phosphate isomerase; along with enolase, aldolase, and triose phosphate isomerase, glucose phosphate isomerase acts as an autoantigen [180]. Hence, in an attempt to increase energy production for cell survival, HIF-1α also produces antigenic targets, thereby promoting autoimmunity. Moreover, HIF-1α also invokes the differentiation of Th0 lymphocytes into Th17 cells, which are important for autoimmune disease development, including RA [181]. CD-25 expression is markedly reduced under hypoxia compared to normoxia [150], suggesting that under hypoxia, T_reg_ cell activity is significantly diminished. Considering these findings, it is reasonable to hypothesize that hypoxic conditions ultimately enhance the risk for inflammatory and autoimmune diseases.

## Conclusions

**Fig. 3:**
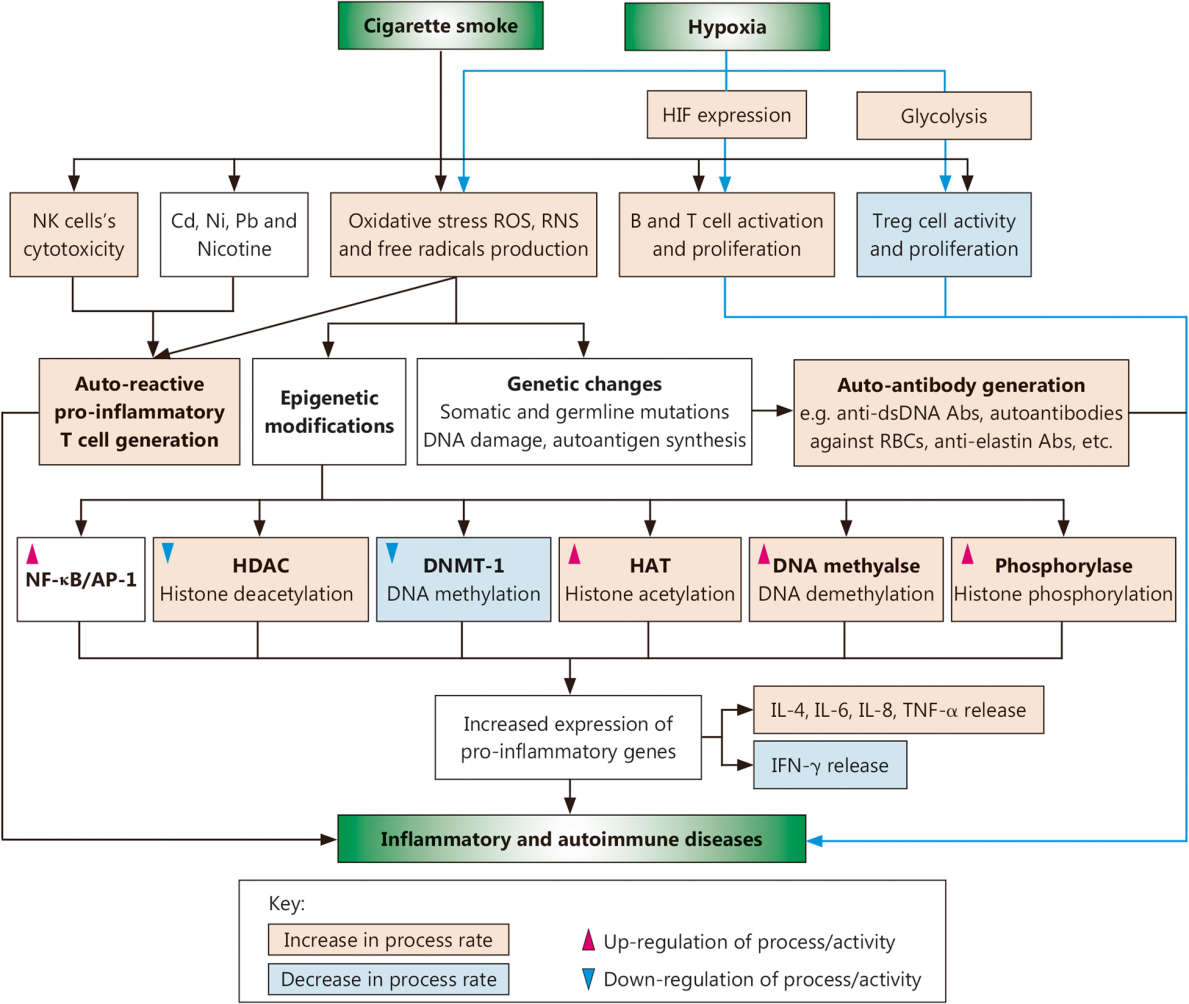
Possible mechanism through which CS together with hypoxic conditions may act as a potent environmental risk factor for inflammatory and autoimmune diseases. CSCs including heavy metals & various immune-modulators and CS-induced oxidative stress enhance the generation of auto-reactive pro-inflammatory T cells, production of autoantibody (e.g. anti-elastin Abs, anti-dsDNA Abs). Oxidative stress induced by CS also causes various genetic and epigenetic changes (increasing the rate of histone acetylation, demethylation and phosphorylation process at the same time decreasing the deacetylation and methylation process rate) which results in increased and sustained expression of pro-inflammatory genes. Similarly, Hypoxia (hypobaric hypoxia and/or cellular hypoxia) by various mechanisms primarily via increasing the HIF expression, oxidative stress and glycolysis rate (Warberg’s effect) also alters the immunological balance (increasing the expression of pro-inflammatory immune-modulators, B and T cell activity and proliferation while decreasing regulates the Treg cell activity and proliferation). Hence, CS in combination with hypoxia acts as a potent environmental risk factor for inflammatory and autoimmune diseases. Abs. Antibodies; AP-1, Activator protein-1; Cd, Cadmium; CS, Cigarette smoke; CSC, Cigarette smoke constituent; DNMT, DNA methyl transferase; HAT, Histone acetylase; HDAC, Histone deacetylase; HIF, Hypoxia-inducible factor; IL, Interleukin; INF, Interferon; NF-κB, Nuclear factor-kappa B; Ni, Nickel; NK, Natural killer cell; Pb, Lead; RNS, Reactive nitrogen species; ROS, Reactive oxygen species; RBC. Red blood cell; TNF, Tumor necrosis factor

In this review, we discussed all of the information reported to date on the association of smoking and hypoxic conditions with inflammatory and autoimmune diseases. The different mechanisms by which smoking and hypoxia may act as potent environmental risk factors for developing and enhancing the severity of inflammatory and autoimmune diseases were related with reference to reported studies. The different mechanisms by which smoking and hypoxic conditions induce inflammatory and autoimmune diseases, including genetic changes (somatic and germline mutations), epigenetic modifications (acetylation/deacetylation, methylation/demethylation and phosphorylation), oxidative stress, auto-antibody formation, CSC heavy metal toxicity and altered immune cell proliferation and pro-inflammatory cytokine production, were analyzed separately. Studies have shown that either of these two environmental factors alone can significantly increase the risk for inflammatory and autoimmune disorders. Although studies showing a direct association of the hypoxic environment (at high altitude) with autoimmune diseases have not yet been reported, there is significant evidence to support that hypoxic conditions result in altered immune system activity, such as increased B and T cell proliferation, reduced T_reg_ cell proliferation/activity, and increased ROS production, resulting in overexpression of pro-inflammatory mediators, such as NF-κB, IL-6, and IL-8. These data suggest that both CS and hypoxia lead to increased oxidative stress and ROS/free radical production, which in turn induces auto-reactive pro-inflammatory T cell production, autoantibody generation (e.g., anti-dsDNA, anti-elastin and anti-RBC autoantibodies), enhanced transcriptional activation/expression of pro-inflammatory mediators (e.g., IL-6, IL-4, IL-8), and reduced expression of IFN-γ by promoting overactivation of NF-κB/APS and various epigenetic modifications. Both CS and hypoxia can also up-regulate expression of auto-immunogenic glycolytic enzymes, reduce T_reg_ cell (immune-suppressive cells) activity and proliferation, and increase B and T cell activation/proliferation via a glycolytic shift (Warberg’s effect), thereby increasing the release of pro-inflammatory mediators. Moreover, these effects are shared by both CS and hypoxia. CS also reduces NK cell cytotoxicity and enhances NF-κB activation due to the heavy metals present in CS, including Cd, Ni and Pb. Furthermore, smoking leverages the hypoxia induced risk of autoimmunity by inhibiting the HIF-1α inhibitor- HDAC. Thus, despite some conflicting data, it is reasonable to hypothesize that smoking and hypoxia (i.e., hypobaric and cellular hypoxia) together may act as a potent environmental risk factor for inflammatory and autoimmune diseases (Fig. 3). Nonetheless, this hypothesis requires further precise studies to explore the association of high-altitude hypoxia with inflammatory and autoimmune diseases. Future studies elucidating the risk of autoimmune disease development in smokers in high-altitude regions, particularly military personnel and mountaineers not acclimatized to high-altitude stresses, may also provide a better understanding for the management of human health in high-altitude regions.

## Abbreviations

AIDS: Acquired immune deficiency syndrome
AIHA: Autoimmune hemolytic anemia
AP-1: Activator protein-1
APS: *Antigen* presentasome
bHLH: *Basic helix-loop-helix*
CBP: CREB-binding *protein*
Cd: Cadmium
CD: Cluster of differentiation
CIA: Collagen-induced arthritis
COPD: Chronic obstructive pulmonary disease
CREB: cAMP-response element-binding *protein*
CS: Cigarette smoke
CSC: Cigarette smoke constituent
DNA: Deoxyribonucleic acid
DNMT: DNA methyl transferase
ERK: Extracellular *signal*-regulated kinase
ESTR: Expanded simple tandem repeat
FIH-1: Factor-inhibiting HIF-1
FLG: Filaggrin
FOXP3: Forkhead box P3
GD: Graves’s disease
GLUT: Glucose transporter
HAT: Histone acetylase
HDAC: Histone deacetylase
HIF: Hypoxia-inducible factor
HIV: Human immunodeficiency virus
HMGB-1: High-mobility group box1
HT: Hashimoto’s thyroiditis
IBD: Inflammatory bowel disease
ICAM-1: Intercellular adhesion molecule-1
IgE: immunoglobulin E
IKK: IκB kinase
IL: Interleukin
INF-γ: Interferon gamma
IκB: Inhibitory kappa B protein
LFA (CD11a/CD18): *Lymphocyte function-associated* antigen-a heterodimer composed of *CD11a* and *CD18*
MHC: Major histocompatibility complex
MMP: Matrix metalloproteinase
MS: Multiple sclerosis
MTS: Mainstream tobacco smoke
NF-κB: Nuclear factor-kappa B
Ni: Nickel
NK: Natural killer cell
NOS: Nitric oxide synthase
Nrf2: Nuclear factor erythroid 2-related factor 2
ODD: Oxygen-dependent degradation
PA: Pernicious anemia
PAS: Per-ARNT-Sim
Pb: Lead
PBA: *Polyclonal B cell* activation
PCAF: p300/(CREB-binding protein)-associated factor
PHD: Prolyl hydroxylase
PTPRN2: Protein-tyrosine phosphatase
RA: Rheumatoid arthritis
RBCs: Red blood cells
RNS: Reactive nitrogen species
RONS: Reactive oxygen and nitrogen species
ROS: Reactive oxygen species
SIRT-1: Rilent information regulator T1
SJ: Sjogren’s syndrome
SLE: Rystemic lupus erythematosus
SNP: Ringle-nucleotide polymorphism
STR: Rimple tandem repeats
T1D: Type-1 diabetes
TIMP-1: Tissue inhibitor of MMP1
TIP60: Tat-interactive protein 60
TNF: Tumor necrosis factor
TPO: Thyroid peroxidase
VEGF: Vascular endothelial growth factor
vHL: von Hippel Lindau
